# A Clinicopathological Analysis of Melanocytic Nevi: A Retrospective Series

**DOI:** 10.3389/fmed.2021.681668

**Published:** 2021-08-10

**Authors:** Panpan Liu, Juan Su, Xuanwei Zheng, Mingliang Chen, Xiang Chen, Jie Li, Cong Peng, Yehong Kuang, Wu Zhu

**Affiliations:** ^1^Department of Dermatology, Xiangya Hospital, Central South University, Changsha, China; ^2^Hunan Key Laboratory of Skin Cancer and Psoriasis, Changsha, China; ^3^Hunan Engineering Research Center of Skin Health and Disease, Changsha, China

**Keywords:** melanocytic nevi, melanocytic diseases, melanoma, diagnosis, misclassification

## Abstract

**Purpose:** Melanocytic nevi are common cutaneous lesions. This study aimed to demonstrate the concordance and discordance between clinical and histopathological diagnoses of melanocytic nevi and the importance of histological evaluation in differentiating malignant lesions from diseases with similar clinical manifestations.

**Patients and Methods:** We studied 4,561 consecutive patients with a clinical diagnosis of melanocytic nevi from 2014 to 2019. We compared the clinical diagnosis with the histopathological diagnosis to establish a histopathological concordance rate and then investigated the effects of clinical characteristics and the reasons for removal on misclassification.

**Results:** Among 4,561 patients who were clinically diagnosed with melanocytic nevi, the overall histopathological concordance rate was 82.11% (3,745 of 4,561 patients), while the histopathological discordance rate was 17.89% (816 of 4,561 patients). The histopathological concordance included 90.25% common acquired melanocytic nevi (3,380 of 3,745 patients) and 9.75% other benign melanocytic neoplasms (365 of 3,745 patients). The most common diagnostic change was to seborrheic keratosis (*n* = 470, 10.30%), followed by basal cell carcinoma (*n* = 64, 1.40%), vascular tumor (*n* = 53, 1.16%), fibroma (*n* = 43, 0.94%), epidermoid cyst (*n* = 34, 0.75%), wart (*n* = 30, 0.66%), melanoma (*n* = 24, 0.53%), Bowen's disease (*n* = 16, 0.35%), squamous cell carcinoma (*n* = 4, 0.09%), keratoacanthoma (*n* = 2, 0.04%), and other neoplasms (*n* = 76, 1.67%). Male sex, old age, location of the lesion, and the reasons for removal have a potential effect on misclassification. The percentages of misclassified lesions on the trunk and limbs and the perineum and buttocks were higher than those in lesions without a change in diagnosis. Importantly, locations of lesions on the head and neck were significantly related to a change in diagnosis to non-melanoma skin cancer, while locations on the hands and feet were significantly related to a change in diagnosis to melanoma. In addition to a typical clinical features, removal due to lesion changes or repeated stimulation was significantly associated with a change in diagnosis to melanoma.

**Conclusions:** Our study emphasizes the clinical differential diagnosis of melanocytic nevi, especially the possibility of malignant tumors. The occurrence of clinical features associated with clinicopathological discordance should raise the clinical suspect and be carefully differentiated from malignant tumors.

## Introduction

Melanocytic nevi are benign tumors of melanocytes; these growths include common acquired melanocytic nevi and other benign melanocytic neoplasms, such as blue nevus, halo nevus, congenital nevomelanocytic nevus, “dysplastic” melanocytic nevus, and Spitz nevus (1–3). Common acquired melanocytic nevi are the most frequent neoplasms. It is unnecessary to remove melanocytic nevi routinely, but they should be removed when any of the following conditions are met: changes in skin lesions, an atypical clinical appearance suspicious for melanoma, cosmetic requirements, or repeated stimulation (4).

In fact, studies have found that common acquired melanocytic nevi are challenging to differentiate in clinical practice from other benign melanocytic neoplasms, such as congenital nevomelanocytic nevus and blue nevus, or even tumors, such as melanoma (5). Moreover, the importance of melanocytic nevi is related to melanoma. A large proportion of melanoma occur in the same area as long-term pre-existing melanocytic nevi (1). Histological examination has shown that approximately 30% of melanoma cases are associated with a residual nevus (6). Therefore, to provide support for clinicians in diagnosing and treating melanocytic nevi, our study focuses on patients with a clinical diagnosis of melanocytic nevi, which are reclassified after a histopathological examination, and assesses whether the clinical characteristics of patients affect the misclassification.

## Patients and Methods

This is a retrospective review of data from 4,561 consecutive patients with a clinical diagnosis of melanocytic nevi over 5 years, from 2014 to 2019, referred to the Department of Dermatology of Xiangya Hospital, Central South University. All patients in this study had undergone an initial clinical diagnosis, excision, and histopathological diagnosis. The reasons for removal of melanocytic nevi included atypical clinical features, changes in skin lesions, cosmetic requirements, or repeated stimulation (e.g., sites of friction or repeated trauma) (4). To ensure accuracy, two independent dermatopathologists reviewed the hematoxylin-and-eosin-stained slides and made a diagnosis. If there is any disagreement among them, another dermatopathologist reviewed the slide, and the three dermatopathologists made the diagnosis together. Clinical data on each case, including the age and sex of the patient and the location of the lesion, were obtained from the patient records. This study was approved by the ethics committees of Xiangya Hospital of Central South University, Changsha, Hunan, China, and informed consent was obtained from all subjects.

The diagnosis and the classification of skin lesions were performed according to Fourth *Edition Dermatology* (edited by Bolognia, J. L.) (7). Histopathological concordance, which means the consistency of clinical and histopathological diagnoses, was assessed for neoplasms clinically diagnosed as melanocytic nevi and histopathologically diagnosed as common acquired melanocytic nevi or other benign melanocytic neoplasms, such as congenital nevomelanocytic nevus, blue nevus, lentigo, “dysplastic” melanocytic nevus, recurrent nevus, Spitz nevus, and halo nevus. Histopathological discordance, which means the inconsistency between clinical and histopathological diagnoses, included clinical diagnosis as melanocytic nevi and histopathological diagnosis as seborrheic keratosis, basal cell carcinoma, vascular tumor, fibroma, epidermoid cyst, wart, melanoma, Bowen's disease, squamous cell carcinoma, keratoacanthoma, and other neoplasms.

The records of 4,561 patients were reviewed to assess demographic and clinical factors, reasons for removal, and histopathological diagnosis. We first compared the clinical diagnosis with the histopathological diagnosis to establish a histopathological concordance rate and then investigated the effect of clinical characteristics and the reasons for removal on misclassification. Statistical analysis was performed using a χ^2^ test and Fisher's exact test. A two-tailed *P* < 0.05 was considered statistically significant. All statistical analyses were performed using the SPSS 23.0 statistical package (IBM SPSS, Chicago, IL, USA).

## Results

### Histopathological Review

**Figure 1:**
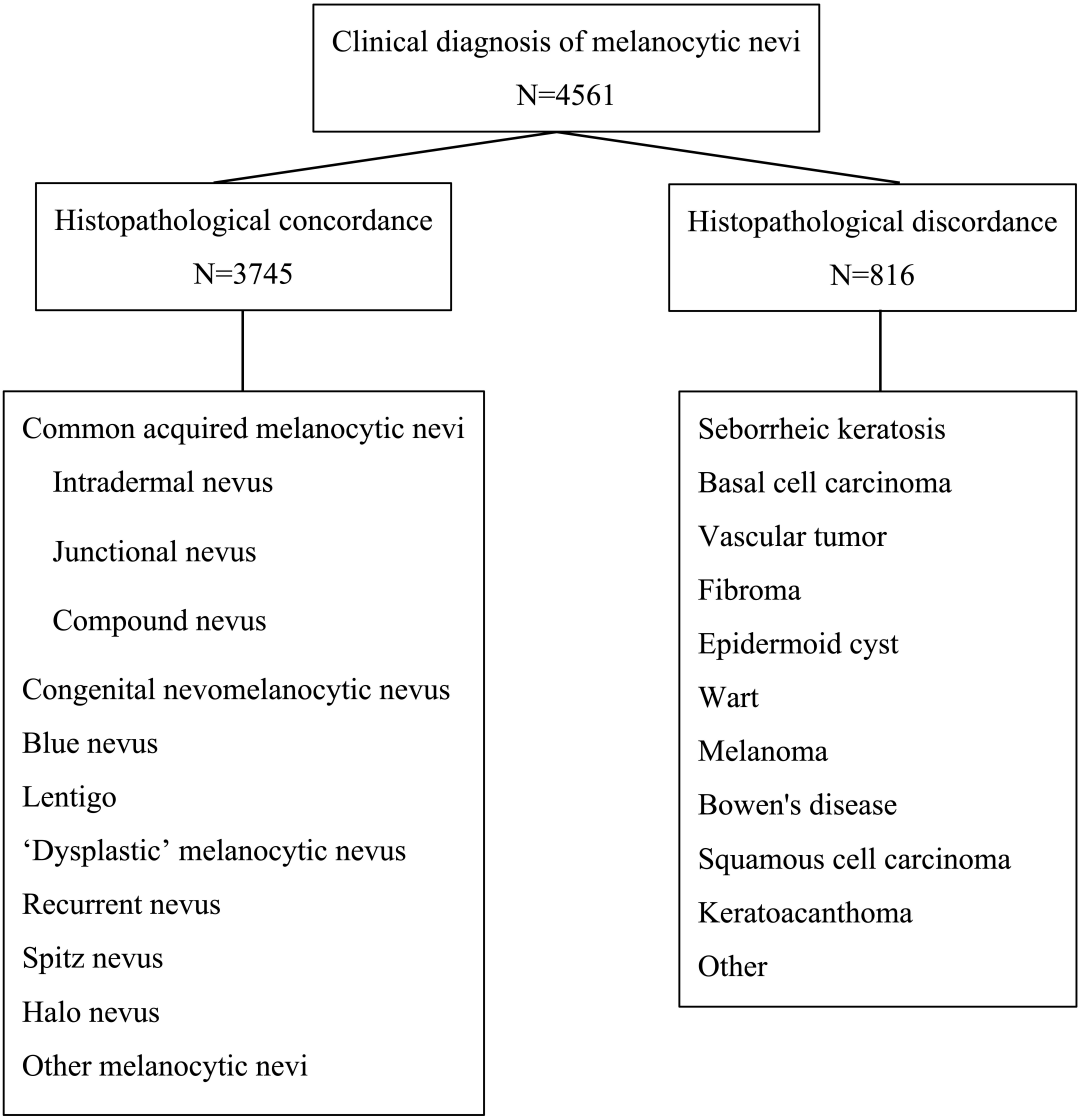
Methodology of the study.

A total of 4,561 patients were clinically diagnosed with melanocytic nevi according to their skin lesions (Figure 1). The characteristics of the patients are listed in Table 1. There were 1,459 males (31.99%) and 3,102 females (68.01%). The age ranged from 2 to 86 years, with a mean age of 31. The patients were divided into four groups based on age: <20, 20–39, 40–59, and ≥60. The most common age range was 20–39 years (54.70%), followed by <20 years (21.75%), while the least common age was ≥60 years (4.71%). The head and neck (66.24%) were the most common sites of involvement, whereas, the hands (1.43%) and the buttocks (0.75%) were the least common sites of involvement. In addition, the most common reasons for removing melanocytic nevi were cosmetic requirements (48.78%) and atypical clinical features (47.18%).

**Table 1:** Clinical characteristics of 3,745 patients with a clinical diagnosis of melanocytic nevi with histopathological concordance.

**Clinical diagnosis of melanocytic nevi with histopathological concordance**																						
**Characteristic**			**Common acquired melanocytic nevi**				**Other benign melanocytic neoplasms**															
			**Intradermal nevus (*N*, %)**	**Junctional nevus (*N*, %)**	**Compound nevus (*N*, %)**		**Congenital nevomelanocytic nevus (*N*, %)**	***P-*value^a^**	**Blue nevus (*N*, %)**	***P-*value^a^**	**Lentigo (*N*, %)**	***P-*value^a^**	**“Dysplastic” melanocytic nevus (*N*, %)**	***P-*value^a^**	**Recurrent nevus (*N*, %)**	***P-*value^a^**	**Spitz nevus (*N*, %)**	***P-*value^a^**	**Halo nevus (*N*, %)**	***P-*value^a^**	**Other melanocytic nevi^b^ (*N*, %)**	***P-*value^a^**
Number	4,561	3,745	2,598	238	544	3,380	114		90		64		39		17		14		12		15	
		82.11%	76.86%	7.04%	16.09%	90.25%	3.04%		2.40%		1.71%		1.04%		0.45%		0.37%		0.32%		0.40%	
Sex																						
Male	1,459	1,099	739	71	171	981	34	0.853	35	0.042	21	0.509	12	0.811	2	0.195	4	1.000	5	0.336	5	0.714
	31.99%	29.35%	28.44%	29.83%	31.43%	29.02%	29.82%		38.89%		32.81%		30.77%		11.76%		28.57%		41.67%		33.33%	
Female	3,102	2,646	1,859	167	373	2,399	80		55		43		27		15		10		7		10	
	68.01%	70.65%	71.56%	70.17%	68.57%	70.98%	70.18%		61.11%		67.19%		69.23%		88.24%		71.43%		58.33%		66.67%	
Age								<0.001		0.009		0.216		0.364		0.625		<0.001		1.000		0.288
Mean	31	27	31	29	28	27	20		31		28		23		23		12		27		23	
Range	2–86	2–80	5–76	2–80	5–76	2–80	6–52		9–79		7–62		8–44		7–51		3–29		13–57		7–51	
<20	992	958	530	54	243	827	62		19		9		13		6		12		3		7	
	21.75%	25.58%	20.40%	22.69%	44.67%	24.47%	54.39%		21.11%		14.06%		33.33%		35.29%		85.71%		25.00%		46.67%	
20–39	2,495	2,250	1,651	144	268	2,063	40		50		46		24		10		2		8		7	
	54.70%	60.08%	63.55%	60.50%	49.26%	61.04%	35.09%		55.56%		71.88%		61.54%		58.82%		14.29%		66.67%		46.67%	
40–59	859	481	379	32	29	440	12		16		8		2		1		0		1		1	
	18.83%	12.84%	14.59%	13.45%	5.33%	13.02%	10.53%		17.78%		12.50%		5.13%		5.88%		0.00%		8.33%		6.67%	
≥60	215	56	38	8	4	50	0		5		1		0		0		0		0		0	
	4.71%	1.50%	1.46%	3.36%	0.74%	1.48%	0.00%		5.56%		1.56%		0.00%		0.00%		0.00%		0.00%		0.00%	
Location								0.068		<0.001		<0.001		<0.001		0.534		<0.001		0.964		<0.001
Head and neck	3,021	2,628	2,067	32	335	2,434	89		38		13		16		16		8		10		4	
	66.24%	70.17%	79.56%	13.45%	61.58%	72.01%	78.07%		42.22%		20.31%		41.03%		94.12%		57.14%		83.33%		26.67%	
Upper limbs	149	118	61	4	27	92	7		15		1		2		1		0		0		0	
	3.27%	3.15%	2.35%	1.68%	4.96%	2.72%	6.14%		16.67%		1.56%		5.13%		5.88%		0.00%		0.00%		0.00%	
Lower limbs	168	109	39	8	34	81	4		7		4		7		0		2		0		4	
	3.68%	2.91%	1.50%	3.36%	6.25%	2.40%	3.51%		7.78%		6.25%		17.95%		0.00%		14.29%		0.00%		26.67%	
Trunk	763	522	375	32	73	480	12		3		10		9		0		1		2		5	
	16.73%	13.94%	14.43%	13.45%	13.42%	14.20%	10.53%		3.33%		15.63%		23.08%		0.00%		7.14%		16.67%		33.33%	
Perineum	108	60	27	10	12	49	0		0		10		1		0		0		0		0	
	2.37%	1.60%	1.04%	4.20%	2.21%	1.45%	0.00%		0.00%		15.63%		2.56%		0.00%		0.00%		0.00%		0.00%	
Hands	65	57	7	21	6	34	0		14		8		1		0		0		0		0	
	1.43%	1.52%	0.27%	8.82%	1.10%	1.01%	0.00%		15.56%		12.50%		2.56%		0.00%		0.00%		0.00%		0.00%	
Feet	253	228	12	131	53	196	2		8		18		3		0		1		0		0	
	5.55%	6.09%	0.46%	55.04%	9.74%	5.80%	1.75%		8.89%		28.13%		7.69%		0.00%		7.14%		0.00%		0.00%	
Buttocks	34	23	10	0	4	14	0		5		0		0		0		2		0		2	
	0.75%	0.61%	0.38%	0.00%	0.74%	0.41%	0.00%		5.56%		0.00%		0.00%		0.00%		14.29%		0.00%		13.33%	
Reason for removal								0.002		0.001		<0.001		0.319		<0.001		0.135		<0.001		0.204
Atypical clinical features	2,152	1,566	1,051	92	218	1,361	67		53		27		20		6		10		12		10	
	47.18%	41.82%	40.45%	38.66%	40.07%	40.27%	58.77%		58.89%		42.19%		51.28%		35.29%		71.43%		100.00%		66.67%	
Changes in skin lesions	30	18	9	0	3	12	0		1		2		0		3		0		0		0	
	0.66%	0.48%	0.35%	0.00%	0.55%	0.36%	0.00%		1.11%		3.13%		0.00%		17.65%		0.00%		0.00%		0.00%	
Cosmetic requirements	2,225	2,015	1,515	80	285	1,880	45		36		23		17		5		4		0		5	
	48.78%	53.81%	58.31%	33.61%	52.39%	55.62%	39.47%		40.00%		35.94%		43.59%		29.41%		28.57%		0.00%		33.33%	
Repeated stimulation	154	146	23	66	38	127	2		0		12		2		3		0		0		0	
	3.38%	3.90%	0.89%	27.73%	6.99%	3.76%	1.75%		0.00%		18.75%		5.13%		17.65%		0.00%		0.00%		0.00%	

a *Demographic comparison was made between the common acquired melanocytic nevi group and other benign melanocytic neoplasms group*.

b *Other melanocytic neoplasms include five spindle cell nevus of Reed, three Becker's nevus, two deep penetrating nevus, one nevus of Ota, one nevus of Ito, and three nevus spilus*.

After a histopathological examination, 3,745 patients were finally diagnosed with melanocytic nevi (overall histopathological concordance rate, 82.11%), while 816 patients clinically diagnosed with melanocytic nevi were reclassified as other diseases (overall histopathological discordance rate 17.89%; Figure 1; Tables 1, 2).

**Table 2:** Histopathological diagnosis distribution of 816 patients whose clinical diagnosis was not congruent with the histopathological diagnosis.

**Histopathological diagnosis**	**Clinical diagnosis of melanocytic nevi**	
	**No. of patients**	**%**
Overall histopathological discordance	816	17.89
Seborrheic keratosis	470	10.30
Basal cell carcinoma	64	1.40
Vascular tumor	53	1.16
Fibroma	43	0.94
Epidermoid cyst	34	0.75
Wart	30	0.66
Melanoma	24	0.53
Bowen's disease	16	0.35
Squamous cell carcinoma	4	0.09
Keratoacanthoma	2	0.04
Other^a^	76	1.67

a *Other include nine granuloma, six sebaceous hyperplasia, six scar, four lichenoid keratosis, one Darier disease, two xanthogranuloma, five dermatitis, three blood blister, two venous lakes, one folliculitis, one cutaneous amyloidosis, one mucinosis, one solar keratosis, one trichilemmal cyst, two lichen sclerosus et atrophicus, four fibrous papule of nose, two Fordyce disease, three hamartomas, one mixed tumor, four trichoepithelioma, four pilomatricoma, two syringoma, one poroma, one hidradenoma, one sebaceoma, one plexiform schwannoma, one xanthoma, three lymphangioma, one clear cell acanthoma, one dermal duct tumor, and one steatocystoma*.

### Histopathological Concordance and Its Clinical Characteristics

The histopathologically concordant cases included common acquired melanocytic nevi (*n* = 3,380, 90.25%) and other benign melanocytic neoplasms (365, 9.75%; Table 1). Common acquired melanocytic nevi were divided into intradermal nevus (*n* = 2,598, 76.86%), junctional nevus (*n* = 238, 7.04%), and compound nevus (544, 16.09%; Table 1). The ratio of males to females was roughly the same in all three type-based groups (overall, 981:2,399; 29.02:70.98%). The most common age range was 20–39 years (61.04%), followed by <20 years (24.47%). The most common sites of involvement for intradermal nevus and compound nevus were the head and neck (79.56 and 61.58%, respectively), followed by the trunk (14.43 and 13.42%, respectively). However, the most common site of involvement in the junctional nevus group was the feet (55.04%), followed by the head and neck (13.45%) and the trunk (13.45%). The main reasons for removing common acquired melanocytic nevi were cosmetic requirements and atypical clinical features. Interestingly, in the junctional nevus group, 27.73% of the lesions were removed because of repeated stimulation, which was significantly higher than the corresponding rates in the intradermal nevus and compound nevus groups (0.89 and 6.99%, respectively).

The other benign melanocytic neoplasms included congenital nevomelanocytic nevus (3.04%), blue nevus (2.40%), lentigo (1.71%), “dysplastic” melanocytic nevus (1.04%), recurrent nevus (0.45%), Spitz nevus (0.37%), halo nevus (0.32%), and other melanocytic nevi (0.40%). We evaluated clinical characteristics that could potentially have an impact on the classification of other benign melanocytic neoplasms. Younger age was significantly associated with the histopathological diagnosis of congenital nevomelanocytic nevus and Spitz nevus, compared with the common acquired melanocytic nevi. The most common age group in the classification of congenital nevomelanocytic nevus and Spitz nevus was <20 years (54.39 and 85.71%, respectively), compared with 20–39 years (61.04%) in patients with a histopathological diagnosis of common acquired melanocytic nevi (Table 1). Furthermore, the location of the lesion was significantly correlated with the diagnosis of blue nevus, lentigo, and “dysplastic” melanocytic nevus (Table 1). The percentages of blue nevus lesions occurring on the upper limbs and hands, lentigo lesions on the feet, and “dysplastic” melanocytic nevus lesions on the lower limbs were higher than those in patients with a histopathological diagnosis of common acquired melanocytic nevi (Table 1). The reasons for removing melanocytic nevi also influenced the classification of congenital nevomelanocytic nevus, blue nevus, lentigo, recurrent nevus, and halo nevus. In the lentigo and recurrent nevus groups, in addition to atypical clinical features, the percentage of lesions removed due to lesion changes and repeated stimulation was significantly higher for lesions histopathologically diagnosed as common acquired melanocytic nevi (Table 1).

### Clinical Characteristics Have a Potential Effect on Misclassification

Histopathological review led to a change in diagnosis in a total of 816 of 4,561 patients (17.89%; Figure 1 and Table 2), including seborrheic keratosis (*n* = 470, 10.30%), basal cell carcinoma (*n* = 64, 1.40%), vascular tumor (*n* = 53, 1.16%), fibroma (*n* = 43, 0.94%), epidermoid cyst (*n* = 34, 0.75%), wart (*n* = 30, 0.66%), melanoma (*n* = 24, 0.53%), Bowen's disease (*n* = 16, 0.35%), squamous cell carcinoma (*n* = 4, 0.09%), keratoacanthoma (*n* = 2, 0.04%), and other neoplasms (*n* = 76, 1.67%).

We evaluated clinical characteristics that could potentially have an impact on misclassification. Among patients with a clinical diagnosis of melanocytic nevi, male sex was significantly related to a change in diagnosis (overall histopathological concordance vs. overall histopathological discordance, *p* < 0.001; Table 3). In addition, old age was also significantly associated with a change in diagnosis (*p* < 0.001; Table 3). The median age of diagnosis of patients with misclassification was 45 years (range, 2–86 years), compared with 27 years (range, 2–80 years) in patients with histopathological concordance. Interestingly, the location of the lesion was significantly correlated with histopathological discordance (*p* < 0.001; Table 3). The percentages of misclassified lesions on the trunk and limbs and the perineum and buttocks were 40.69 and 7.11%, respectively, compared with 20.00 and 2.22%, respectively, in lesions without a change in diagnosis (Table 3). The reasons for removing melanocytic nevi also influenced the change in diagnosis. The proportion of misclassified lesions removed due to atypical clinical features was 71.81%, compared with 41.82% among lesions with histopathological concordance (*p* < 0.001; Table 3). Therefore, sex, age, location of the lesion, and reasons for removal all have a potential effect on misclassification.

**Table 3:** Clinical characteristics of patients with histopathological concordance or histopathological discordance.

**Clinical diagnosis of melanocytic nevi**																
**Characteristic**			**Histopathological discordance**													
		**Overall histopathological concordance (*N*, %)**	**Overall histopathological discordance (*N*, %)**	***P*-value^a^**	**Seborrheic keratosis (*N*, %)**	***P*-value^a^**	**Basal cell carcinoma (*N*, %)**	***P*-value^a^**	**Melanoma (*N*, %)**	***P*-value^a^**	**Bowen's disease (*N*, %)**	***P*-value^a^**	**Squamous cell carcinoma (*N*, %)**	***P*-value^a^**	**Keratoacanthoma (*N*, %)**	***P*-value^a^**
Number	4,561	3,745	816		470		64		24		16		4		2	
		82.11%	17.89%		10.30%		1.40%		0.53%		0.35%		0.09%		0.04%	
Sex				<0.001		<0.001		<0.001		0.077		<0.001		0.721		1.000
Male	1,459	1,099	360		201		37		11		13		2		1	
	31.99%	29.35%	44.12%		42.77%		57.81%		45.83%		81.25%		50.00%		50.00%	
Female	3,102	2,646	456		269		27		13		3		2		1	
	68.01%	70.65%	55.88%		57.23%		42.19%		54.17%		18.75%		50.00%		50.00%	
Age				<0.001		<0.001		<0.001		<0.001		<0.001		<0.001		0.007
Mean	31	27	45		48		55		50		43		49		63	
Range	2–86	2–80	2–86		3–86		21–80		17–73		25–69		34–64		50–76	
<60	4,346	3,689	657		376		39		14		12		2		1	
	95.29%	98.50%	80.51%		80.00%		60.94%		58.33%		75.00%		50.00%		50.00%	
≥60	215	56	159		94		25		10		4		2		1	
	4.71%	1.50%	19.49%		20.00%		39.06%		41.67%		25.00%		50.00%		50.00%	
Location				<0.001		<0.001		0.001		<0.001		<0.001		0.723		1.000
Head and neck	3,021	2,628	393		218		59		1		1		4		2	
	66.24%	70.17%	48.16%		46.38%		92.19%		4.17%		6.25%		100.00%		100.00%	
Trunk and limbs	1,081	749	332		228		3		6		3		0		0	
	23.70%	20.00%	40.69%		48.51%		4.69%		25.00%		18.75%		0.00%		0.00%	
Perineum and buttocks	141	83	58		18		2		2		10		0		0	
	3.09%	2.22%	7.11%		3.83%		3.13%		8.33%		62.50%		0.00%		0.00%	
Hands and feet	318	285	33		6		0		15		2		0		0	
	6.97%	7.61%	4.04%		1.28%		0.00%		62.50%		12.50%		0.00%		0.00%	
Reason for removal				<0.001		<0.001		<0.001		<0.001		0.018		0.094		0.261
Atypical clinical features	2152	1566	586		328		54		16		13		4		2	
	47.18%	41.82%	71.81%		69.79%		84.38%		66.67%		81.25%		100.00%		100.00%	
Changes in skin lesions	30	18	12		3		4		4		0		0		0	
	0.66%	0.48%	1.47%		0.64%		6.25%		16.67%		0.00%		0.00%		0.00%	
Cosmetic requirements	2225	2015	210		139		6		1		3		0		0	
	48.78%	53.81%	25.74%		29.57%		9.38%		4.17%		18.75%		0.00%		0.00%	
Repeated stimulation	154	146	8		0		0		3		0		0		0	
	3.38%	3.90%	0.98%		0.00%		0.00%		12.50%		0.00%		0.00%		0.00%	

a *Demographic comparison was made between the overall histopathological concordance group and histopathological discordance group*.

In further detail, male sex was significantly associated with a change in diagnosis to seborrheic keratosis, basal cell carcinoma, Bowen's disease, and wart (*p* < 0.001; Table 3 and Supplementary Table 1). However, sex had no effect on the change in diagnosis to melanoma (Table 3). Old age was significantly correlated with changes in diagnosis to seborrheic keratosis, basal cell carcinoma, melanoma, Bowen's disease, squamous cell carcinoma, keratoacanthoma, epidermoid cyst, and wart (*p* < 0.001; Table 3 and Supplementary Table 1). Furthermore, lesions on the trunk and limbs were significantly associated with a change in diagnosis to seborrheic keratosis (48.51%), lesions on the head and neck were significantly associated with a change in diagnosis to basal cell carcinoma (92.19%), lesions on the hands and feet were significantly associated with a change in diagnosis to melanoma (62.50%), and lesions on the perineum and buttocks were significantly associated with changes in diagnosis to Bowen's disease (62.50%) and wart (26.67%), compared with 20.00, 70.17, 7.61, and 2.22%, respectively, in patients with histopathological concordance (Table 3 and Supplementary Table 1). More importantly, in addition to atypical clinical features, the percentages of lesions removed due to lesion changes (16.76%) and repeated stimulation (12.50%) were significantly higher among lesions with a change in diagnosis to melanoma than among lesions with a histopathological concordance (0.48 and 3.90%, respectively; Table 3). Therefore, clinical characteristics and reasons for removal have a potential impact on misclassification.

## Discussion

Melanocytic nevi are the most common benign neoplasms of the skin and are also the most easily misdiagnosed skin disease (5). Our study collected excised lesions clinically diagnosed as melanocytic nevi in a Chinese population over 5 consecutive years. The results showed that the consistency of the clinical and histopathological diagnoses of melanocytic nevi was 82.11% after the histopathological examination. Among them, 90.25% were common acquired melanocytic nevi, and 9.75% were other benign melanocytic neoplasms, such as congenital nevomelanocytic nevi, blue nevi, and lentigo. More importantly, 17.89% of patients were reclassified as having other diseases after the histopathological examination. The most common diagnostic change was to seborrheic keratosis, followed by basal cell carcinoma. Overall, 24 (0.53%) patients were reclassified as having melanoma. In addition, clinical characteristics, such as sex, age, and location of the lesion, and the reasons for removal have a potential impact on misclassification.

Morphologically, common acquired melanocytic nevi are challenging to distinguish from other benign melanocytic neoplasms (8). Our study found that, among the cases with histopathological concordance, 9.75% were other types of benign melanocytic neoplasms. In addition, the results showed that younger age was significantly associated with the histopathological diagnosis of congenital nevomelanocytic nevus and Spitz nevus, compared with common acquired melanocytic nevi. The reason for the clinical significance of congenital nevomelanocytic nevus (CMN) is the risk of malignancy (9). The malignancy potential of CMN is well-characterized in congenital melanocytic giant nevi (10). However, the possibility of melanoma development has also been clearly verified for medium and small nevi (11). Spitz nevus is predominantly observed in children and adolescents. In children, a Spitz nevus presents as isolated, domed nodules with a smooth surface and a bright red to brown color. In adults, a Spitz nevus is usually dark in color with brown to black papules, nodules, and nodes (12–14). Because of the difficulty of the clinical diagnosis of Spitz nevus, histopathological examination is necessary (15, 16). Therefore, patients should be asked for a detailed medical history, and lesions in younger patients should be differentiated from congenital nevomelanocytic nevus and Spitz nevus. “Dysplastic” melanocytic nevus, also known as atypical melanocytic nevus, is unusually large and variable in form and shows atypical asymmetry, size, borders, and coloration (7). Moreover, “dysplastic” melanocytic nevus is challenging to distinguish from common acquired melanocytic nevi and melanoma (17). Importantly, it has been reported that melanoma can develop in “dysplastic” melanocytic nevi with a probability of 1:200 to 1:500 and that the presence of several “dysplastic” melanocytic nevi increases the melanoma risk (3). Our data found that the percentage of “dysplastic” melanocytic nevus lesions occurring on the lower limbs was higher than that in patients with a histopathological diagnosis of common acquired melanocytic nevi. Therefore, lesions on the lower limbs with atypical clinical features should be given a detailed physical examination and carefully differentiated from common acquired melanocytic nevi.

In this study, 17.89% of lesions were reclassified as other diseases after the histopathological examination. The most common diagnostic change was seborrheic keratosis, a common benign epidermal tumor (18). Seborrheic keratosis is generally a roundish, scaly, reddish to brownish lesion; it is most common in individuals over 50 years old (19, 20). Clinically, seborrheic keratosis can mimic the appearance of melanocytic tumors (21). Our study found that male sex, older age, and location of the lesions on the trunk and limbs have a potential impact on the misclassification of seborrheic keratosis. Thus, skin lesions with the above-mentioned characteristics should be differentiated from seborrheic keratosis. More importantly, after the histopathological examination, 1.89% of patients were reclassified as having non-melanoma skin cancer (NMSC), including 64 with basal cell carcinoma, 16 with Bowen's disease, four with squamous cell carcinoma, and two with keratoacanthoma. NMSC is the most common human cancer, and sun exposure is an important risk factor for this disease (22). In addition, it has been reported that the incidence of NMSC is higher in men than in women, and 80% of cases occur in people aged 60 years and older (23). Basal cell carcinomas are usually small and have a translucent or pearly appearance (24, 25). Approximately 80% of all basal cell carcinomas occur on the head and neck. Unlike basal cell carcinomas, squamous cell carcinomas can have precursor lesions, such as actinic keratosis and Bowen's disease (26), and typically develop on sun-exposed sites. These studies support our findings. Our study found that male sex was significantly associated with changes in diagnosis to basal cell carcinoma and Bowen's disease. Old age and sun-exposed sites (head and neck) have a potential impact on the misclassification of NMSC, except for Bowen's disease. Lesions on the perineum and buttocks were significantly related to a change in diagnosis to Bowen's disease. Therefore, melanocytic nevi-like lesions in elderly and/or male patients and on sun-exposed skin should be carefully differentiated from basal cell carcinoma and squamous cell carcinoma, and lesions on the perineum and buttocks should be differentiated from Bowen's disease.

The most important task in the diagnosis of melanocytic nevi is to differentiate these lesions from melanoma. We found that 24 patients were reclassified as having melanoma after the histopathological examination. Sex had no effect on misclassification. However, older age and lesions on the hands and feet were significantly related to a change in diagnosis to melanoma, which might be related to the characteristics of melanoma in China. The incidence of cutaneous melanoma is rising faster than that of any other solid tumor (27, 28). Superficial spreading melanoma is the most common type of cutaneous melanoma in Caucasians (29). However, acral melanoma is a common subtype of melanoma in Chinese patients, while it is rare in Caucasian patients (30–33). Melanoma can develop from pre-existing nevi in approximately 20–40% of cases (34). Early diagnosis is the key to improving the survival rate (35). The ABCDE rules (asymmetry, border irregularity, color variegation, diameter, and evolution) are useful for the early identification of melanoma (36). In addition, dermatoscopy improves diagnostic accuracy, particularly in the differential diagnosis between benign and malignant melanocytic tumors. Furthermore, the results showed that, in addition to atypical clinical features, the percentage of lesions removed due to lesion changes and repeated stimulation was significantly higher in lesions whose diagnosis was changed to melanoma than in lesions with histopathological concordance. Therefore, our study shows that melanocytic nevi should be differentiated from melanoma in elderly patients when the lesions are in load-bearing and friction-prone sites or in the event of changes or repeated stimulation.

In summary, our data demonstrate that histopathological review results in a change in diagnosis in 17.89% of patients with the clinical diagnosis of melanocytic nevi. In addition, clinical characteristics have a potential impact on misclassification. In addition to atypical clinical manifestations, lesions in elderly patients, lesions in sun-exposed, load-bearing, or friction-prone locations, and lesions with changes or repeated stimulation should raise the clinical suspect and be carefully differentiated from malignant tumors.

## Data Availability Statement

The original contributions presented in the study are included in the article/Supplementary Material, further inquiries can be directed to the corresponding author/s.

## Ethics Statement

The studies involving human participants were reviewed and approved by the ethics committees of Xiangya hospital of Central South University, Changsha, Hunan, China. Written informed consent to participate in this study was provided by the participants' legal guardian/next of kin.

## Author Contributions

PL performed the study design, data analysis, and manuscript writing. XZ, MC, XC, JL, and CP contributed to data collection and validation. YK, MC, and XC performed the clinical diagnosis and samples collection. JS, YK, and WZ were clinical experts and performed the manuscript revision. All authors read and approved the final version of the manuscript.

## Conflict of Interest

The authors declare that the research was conducted in the absence of any commercial or financial relationships that could be construed as a potential conflict of interest.

## Publisher's Note

All claims expressed in this article are solely those of the authors and do not necessarily represent those of their affiliated organizations, or those of the publisher, the editors and the reviewers. Any product that may be evaluated in this article, or claim that may be made by its manufacturer, is not guaranteed or endorsed by the publisher.
