# Supplementary material for: A Clinicopathological Analysis of Melanocytic Nevi: A Retrospective Series
Source: Front Med (Lausanne). 2021 Aug 10;8:681668. doi: 10.3389/fmed.2021.681668 (PMC8383488; doi:10.3389/fmed.2021.681668)
Supplement: Supplementary file 1 [file Table_1.pdf]

# *Supplementary Material*

## 1 Supplementary Figures and Tables

**Table S1. Clinical characteristics of patients with the histopathological concordance or histopathological discordance**

| | | Clinical diagnosis of melanocytic nevi | | | | | | | | | | | | |
|---|---|---|---|---|---|---|---|---|---|---|---|---|---|---|
| | | | Histopathological discordance | | | | | | | | | | | |
| Characteristic | | Overall histopathological concordance (N, %) | Overall histopathological discordance (N, %) | P value[a] | Vascular tumor (N, %) | P value[a] | Fibroma (N, %) | P value[a] | Epidermoid cyst (N, %) | P value[a] | Wart (N, %) | P value[a] | Other [b] (N, %) | P value[a] |
| Number | 4561 | 3745 | 816 | | 53 | | 43 | | 34 | | 30 | | 76 | |
| | | 82.11% | 17.89% | | 6.50% | | 81.13% | | 79.07% | | 88.24% | | 253.33% | |
| Sex | | | | <0.001 | | 0.302 | | 0.59 | | 0.258 | | <0.001 | | 0.008 |
| Male | 1459 | 1099 | 360 | | 19 | | 11 | | 13 | | 19 | | 33 | |
| | 31.99% | 29.35% | 44.12% | | 35.85% | | 25.58% | | 38.24% | | 63.33% | | 43.42% | |
| Female | 3102 | 2646 | 456 | | 34 | | 32 | | 21 | | 11 | | 43 | |
| | 68.01% | 70.65% | 55.88% | | 64.15% | | 74.42% | | 61.76% | | 36.67% | | 56.58% | |
| Age | | | | <0.001 | | 0.061 | | 0.293 | | <0.001 | | <0.001 | | <0.001 |
| Mean | 31 | 27 | 45 | | 34 | | 37 | | 45 | | 37 | | 39 | |
| Range | 2-86 | 2-80 | 2-86 | | 2-66 | | 14-66 | | 16-81 | | 4-69 | | 4-74 | |
| <60 | 4346 | 3689 | 657 | | 50 | | 41 | | 26 | | 26 | | 70 | |
| | 95.29% | 98.50% | 80.51% | | 94.34% | | 95.35% | | 76.47% | | 86.67% | | 92.11% | |
| >=60 | 215 | 56 | 159 | | 3 | | 2 | | 8 | | 4 | | 6 | |
| | 4.71% | 1.50% | 19.49% | | 5.66% | | 4.65% | | 23.53% | | 13.33% | | 7.89% | |
| Location | | | | <0.001 | | <0.001 | | <0.001 | | 0.039 | | <0.001 | | 0.977 |
| Head and neck | 3021 | 2628 | 393 | | 13 | | 8 | | 21 | | 13 | | 53 | |
| | 66.24% | 70.17% | 48.16% | | 24.53% | | 18.60% | | 61.76% | | 43.33% | | 69.74% | |
| Trunk and limbs | 1081 | 749 | 332 | | 31 | | 27 | | 11 | | 7 | | 16 | |
| | 23.70% | 20.00% | 40.69% | | 58.49% | | 62.79% | | 32.35% | | 23.33% | | 21.05% | |
| Perineum and Buttocks | 141 | 83 | 58 | | 7 | | 7 | | 2 | | 8 | | 2 | |
| | 3.09% | 2.22% | 7.11% | | 13.21% | | 16.28% | | 5.88% | | 26.67% | | 2.63% | |
| Hands and Feet | 318 | 285 | 33 | | 2 | | 1 | | 0 | | 2 | | 5 | |
| | 6.97% | 7.61% | 4.04% | | 3.77% | | 2.33% | | 0.00% | | 6.67% | | 6.58% | |
| Reason for removal | | | | <0.001 | | <0.001 | | 0.004 | | 0.001 | | <0.001 | | 0.001 |
| Atypical clinical | 2152 | 1566 | 586 | | 38 | | 28 | | 26 | | 27 | | 50 | |
| features | 47.18% | 41.82% | 71.81% | | 71.70% | | 65.12% | | 76.47% | | 90.00% | | 65.79% | |
| Changes in skin lesions | 30 | 18 | 12 | | 0 | | 1 | | 0 | | 0 | | 0 | |
| | 0.66% | 0.48% | 1.47% | | 0.00% | | 2.33% | | 0.00% | | 0.00% | | 0.00% | |
| Cosmetic requirements | 2225 | 2015 | 210 | | 12 | | 14 | | 8 | | 3 | | 24 | |
| | 48.78% | 53.81% | 25.74% | | 22.64% | | 32.56% | | 23.53% | | 10.00% | | 31.58% | |
| Repeated stimulation | 154 | 146 | 8 | | 3 | | 0 | | 0 | | 0 | | 2 | |
| | 3.38% | 3.90% | 0.98% | | 5.66% | | 0.00% | | 0.00% | | 0.00% | | 2.63% | |

[a] Demographic comparison was made between the overall histopathological concordance group and histopathological discordance group.

[b] Other include 9 granuloma, 6 sebaceous hyperplasia, 6 scar, 4 lichenoid keratosis, 1 darier disease, 2 xanthogrannloma, 5 dermatitis, 3 blood blister, 2 venous lakes, 1 folliculitis, 1 cutaneous amyloidosis, 1 mucinosis, 1 solar keratosis, 1 trichilemmal cyst, 2 lichen sclerosus et atrophicus, 4 fibrous papule of nose, 2 fordyce disease, 3 hamartomas, 1 mixed tumor, 4 trichoepithelioma, 4 pilomatricoma, 2 syringoma, 1 poroma, 1 hidradenoma, 1 sebaceoma, 1 plexiform schwannoma, 1 xanthoma, 3 lymphangioma, 1 clear cell acanthoma, 1 dermal duct tumor, and 1 steatocystoma.
